# Why thorough open data descriptions matters more than ever in the age of AI: opportunities for cardiovascular research

**DOI:** 10.1093/ehjdh/ztae061

**Published:** 2024-08-08

**Authors:** Sandy Engelhardt

**Affiliations:** Department of Cardiology, Angiology and Pneumology, Heidelberg University Hospital, Im Neuenheimer Feld 410, D-69120 Heidelberg, Germany; Department of Cardiac Surgery, Heidelberg University Hospital, Im Neuenheimer Feld 410, D-69120 Heidelberg, Germany; German Centre for Cardiovascular Research (DZHK), Partner Site Heidelberg/Mannheim, Germany; AI Health Innovation Cluster (AIH), Heidelberg, Germany; Informatics for Life Institute, Heidelberg, Germany

## Introduction

The quality of trained AI algorithms and the validity of the conclusions are highly dependent on the quality, size, and properties of the data sets used for model training and testing. Systematic errors or prejudices in AI algorithms that can occur lead to unfair outcomes and are referred to as bias, often disadvantaging certain groups based on characteristics such as race, gender, age, or socioeconomic status.

Data bias in medicine occurs when the training data do not adequately represent the population. For example, AI algorithms developed for predicting heart disease might underdiagnose women if trained primarily on male-dominated data sets, reflecting historical underrepresentation of women in clinical studies.^1^ Furthermore, algorithm might not be sufficiently well tested when certain groups are not present in the test set.^2^ Ensuring that data sets are representative of the target population is therefore key for scientific progress and usability of AI in medicine to increase fairness, accuracy, and reliability in healthcare applications. This includes balancing data sets across different demographics to avoid skewed results, for which rigorous documentation of such important cohort properties are a prerequisite.

Models generalize better across different patient populations and medical conditions, which can be facilitated if data from different sources is openly accessible under ethical and legal compliance. Sharing data openly allows for the identification and mitigation of biases, leading consequently to potentially fairer and more accurate models. They further allow independent validation and benchmarking of models against each other, increasing their reliability and robustness in clinical settings. One AI example that was rigorously trained and tested on diverse open data is the nnUnet framework,^3^ which is now regarded as a standard method for many 2D and 3D medical image segmentation tasks.

As creating data sets and curating data sets in the medicine require a lot of effort, more revenue should be paid to authors who undertake this mission. The European Heart Journal—Digital Health now supports this endeavour by introducing a novel category called ‘Data Paper’. Data Papers are designed to facilitate data reuse and provide credit to those who share data. Researchers are enabled to describe their data sets without needing to include analysis or interpretation. This approach helps increase the visibility and usability of the data, the identification of bias, encouraging reuse by other researchers and crediting providers by citations. Ideally, such papers include thorough statistics on data distributions within patient (sub-)groups. This is a particular important advance for cardiovascular research, as the availability of open data sets in this research field is still limited in comparison with the complex and pressing research questions that need to be addressed given that cardiovascular diseases are the leading cause of death globally.

The FAIR principles established in 2016^4^ provide recommendations for the management and stewardship of data to ensure that data is *Findable*, *Accessible*, *Interoperable*, and *Reusable*. In the context of open data sharing and AI, the FAIR principles are particularly relevant to maximize the utility, reliability, and impact of shared medical data.

In particular, these principles define a series of concrete instructions how data should be made available. For example, data should be assigned a unique and persistent identifier so it can be easily located. Comprehensive metadata should be provided to enhance the discoverability of data through search engines and data catalogues. Comprehensive metadata detailing the origin, collection methods, and any transformations applied to the data enhance its reliability and utility for further research. Data should be indexed in searchable resources to facilitate easy retrieval.

Data should be made available, ideally through open-access repositories that ensure long-term accessibility and availability. When necessary, access to data should be controlled via clear and standardized authentication and authorization procedures to ensure security and compliance with legal and ethical standards. Data should be accompanied by clear usage licenses that specify how it can be reused, ensuring legal clarity and encouraging reuse. Data should be stored in commonly accepted and standardized formats to facilitate integration with other data sets and tools.

Data should be of high quality, well-documented, and relevant to ensure they meet the needs of future users and applications. For example, if manually created annotations are provided, it should be specified who has created these annotations and whether it is a calculated mean over all annotations if several experts where involved. Employing standardized vocabularies and ontologies ensures that data can be consistently interpreted and integrated across different systems. This could entail widely understood descriptions of patient demographics.

## Discussion

Open data sets in cardiology have been instrumental in driving several advancements and innovations. Here are a few notable success stories that originated from the use of these open data sets: the Automated Cardiac Diagnosis Challenge (ACDC) data set has been widely used to develop and benchmark state-of-the-art algorithms for the segmentation of the left and right ventricles and myocardium. These algorithms have significantly improved the accuracy and efficiency of cardiac MRI analysis, aiding in the diagnosis and management of various heart conditions.^5^ The summarizing publication was cited more than 1500 times and according to a recent study, it was equivalent mentioned as often in research papers.^6^ Further mentions deserve similar cardiac MRI datasets like M&M^7^ and M&M2.^8^

Physionet is a multicentre resource that was established in 1999, under the auspices of the National Center for Research Resources, National Institutes of Health.^9^ It hosts various data sets, such as the MIMIC-III (Medical Information Mart for Intensive Care) data set, which is a large, single-centre database comprising information relating to 40 000 patients admitted to critical care units at a large tertiary care hospital;^10^ the newer MIMICS-IV data set^11^ builds on top of that and incorporates contemporary data. Both databases are highly used resources. Zheng *et al*.^12^ released 10 646 patient ECGs to the research community, now cited more than 300 times. Despite these encouraging developments, a recent review on machine learning and AI in cardiovascular research reports that only 8% of the 215 investigated papers do share data.^13^

It is known that publicly available data sets have a measurable impact on the citation rates of research articles. After analysing 10 000 studies in the biomedical field, the study by Piwowar and Vision^14^ found that articles with publicly available data sets received significantly more citations than those without accessible data. This effect persisted even after controlling for various factors such as the journal’s impact factor, the number of authors, and the previous publication record of the authors.

This underscores the importance of open data sharing in enhancing the visibility and impact of scientific research. We should advocate for continued efforts to promote data sharing practices and rigorous documentation to benefit the scientific community and advance knowledge dissemination considering that the age of very data-hungry AI algorithms has just begun with the advent of foundation models, e.g. for cardiac computed tomography.^15^ and echocardiography.^16^

## Data Availability

There are no new data associated with this article.
